# Green and Black Cardamom in a Diet-Induced Rat Model of Metabolic Syndrome

**DOI:** 10.3390/nu7095360

**Published:** 2015-09-11

**Authors:** Maharshi Bhaswant, Hemant Poudyal, Michael L. Mathai, Leigh C. Ward, Peter Mouatt, Lindsay Brown

**Affiliations:** 1 Centre for Chronic Disease Prevention & Management, College of Health and Biomedicine, Victoria University, Melbourne 3021, Australia; E-Mails: cmaharshi@gmail.com (M.B.); michael.mathai@vu.edu.au (M.L.M.); 2 School of Health and Wellbeing, University of Southern Queensland, Toowoomba 4350, Australia; 3 Department of Diabetes, Endocrinology and Nutrition, Graduate School of Medicine and the Hakubi Center for Advanced Research, Kyoto University, Kyoto 606-8507, Japan; E-Mail: hpoudyal@kuhp.kyoto-u.ac.jp; 4 School of Chemistry and Molecular Biosciences, The University of Queensland, St Lucia 4072, Australia; E-Mail: l.ward@uq.edu.au; 5 Southern Cross Plant Science, Analytical Research Laboratories, Southern Cross University, East Lismore 2480, Australia; E-Mail: Peter.Mouatt@scu.edu.au

**Keywords:** cardamom, obesity, hypertension, metabolic syndrome, rats

## Abstract

Both black (B) and green (G) cardamom are used as flavours during food preparation. This study investigated the responses to B and G in a diet-induced rat model of human metabolic syndrome. Male Wistar rats were fed either a corn starch-rich diet (C) or a high-carbohydrate, high-fat diet with increased simple sugars along with saturated and *trans* fats (H) for 16 weeks. H rats showed signs of metabolic syndrome leading to visceral obesity with hypertension, glucose intolerance, cardiovascular remodelling and nonalcoholic fatty liver disease. Food was supplemented with 3% dried B or G for the final eight weeks only. The major volatile components were the closely related terpenes, 1,8-cineole in B and α-terpinyl acetate in G. HB (high-carbohydrate, high-fat + black cardamom) rats showed marked reversal of diet-induced changes, with decreased visceral adiposity, total body fat mass, systolic blood pressure and plasma triglycerides, and structure and function of the heart and liver. In contrast, HG (high-carbohydrate, high-fat + green cardamom) rats increased visceral adiposity and total body fat mass, and increased heart and liver damage, without consistent improvement in the signs of metabolic syndrome. These results suggest that black cardamom is more effective in reversing the signs of metabolic syndrome than green cardamom.

## 1. Introduction

Spices are used to flavour foods but they may also be effective as functional foods to improve health or decrease the risk of disease [1,2,3]. In particular, spices may decrease metabolic syndrome, defined as the cluster of obesity, hypertension, diabetes and non-alcoholic fatty liver disease [4]. These metabolic perturbations lead to chronic changes in the structure and function of the heart, liver, kidneys and pancreas [5]. The prevalence of metabolic syndrome is high in both developing and developed countries including the USA (34%), India (25.6%), Kuwait (24.8%) and Australia (22.1%) [6,7,8,9,10].

Cardamom is a well-known spice with green (*Elettaria cardamomum* Maton) and black (*Amomum subulatum* Roxburgh) varieties, both in the family Zingiberaceae, used in culinary and traditional medicine practices. Black cardamom is grown in the north-eastern Indian state of Sikkim as well as in neighbouring Nepal and Bhutan [10] while green cardamom is grown in the southern Indian states of Tamil Nadu, Kerala and Karnataka [11] with Guatemala as the other major source. Dry pods of cardamom contain volatile oils, phenolic acids, lipids and sterols [10,11]. Both black and green cardamom contain terpenes in the essential oils, with 1,8-cineole and α-terpineol found in black cardamom and α-terpinyl acetate and 1,8-cineole in green cardamom [10,11].

Green cardamom has been used since the 4th century BC by Indian Ayurvedic practitioners and ancient Greek and Roman physicians for the treatment of indigestion, bronchitis, asthma and constipation, and to stimulate appetite in anorexia [12,13,14]; other indications include diarrhoea, dyspepsia, epilepsy, hypertension, cardiovascular diseases, ulcers, gastro-intestinal disorders and vomiting [15,16,17]. Similarly, black cardamom is used by Ayurvedic and Unani practitioners for many ailments including indigestion, vomiting, rectal diseases, dysentery, liver congestion, gastrointestinal disorders and genitourinary complaints [14,18].

Rats fed with high-carbohydrate, high-fat diet for eight weeks developed visceral adiposity, impaired glucose tolerance with increased plasma insulin concentrations, increased systolic blood pressure, structural damage to the heart and liver and elevated plasma lipid concentrations [19]. Therefore, in this study, we have compared the cardiovascular, liver and metabolic responses to green and black cardamom in a high-carbohydrate, high-fat diet-fed rat model of human metabolic syndrome [19]. These measurements included systolic blood pressure, echocardiography, vascular reactivity, cardiac collagen deposition, stiffness, plasma biochemistry and histology for structural changes on heart and liver. We report that addition of black cardamom to the diet improved the signs of metabolic syndrome much more effectively than green cardamom. Further, green cardamom may worsen heart and liver structure.

## 2. Experimental Section

### 2.1. Analysis of Green Cardamom and Black Cardamom

100 mg of black or green cardamom was extracted in 3 mL of 100% ethanol by sonication for 10 min. After centrifugation, an aliquot of the supernatant was transferred to a vial and injected into a HP 6890 GC and 5973 MS (Agilent Technologies, Mulgrave, Victoria, Australia). The analysis was performed using on a HP-5MS GC column (Agilent 19091S-433), 30 m × 0.25 µm, with a flow rate of 0.9 mL/min helium at an average velocity of 35 cm/s. The oven settings were an initial 50 °C held for 5 min, with a ramp of 10 °C per minute up to 250 °C, a total run time of 30 min. Inlet temperature was 250 °C with an injection of 1 µL and split ratio of 50:1. MS settings were EM voltage 71, source 230 and quadrupole 150, with a scan for masses between 35 and 350 amu. Constituents were identified by comparison of peak MS spectra with GC MS libraries of NIST, Adams and Wiley with threshold match of >95%. Powdered black and green cardamom were analysed for protein, fat, total carbohydrates and energy value by Symbio Alliance, Brisbane, Queensland, Australia.

### 2.2. Animals and Diets

All experimental protocols were approved by the Animal Ethics Committee of the University of Southern Queensland under the guidelines of the National Health and Medical Research Council of Australia (Ethic Approval Number: 13REA005). The experimental group consisting of 72 male Wistar rats (9–10 weeks old; weighing 335–340 g) was individually housed in a temperature-controlled room under 12-h light/dark cycle environment with excess food and water at the University of Southern Queensland animal house. Rats were randomly divided into six experimental groups (*n* = 12 each) and fed with corn starch (C), corn starch + black cardamom (CB), corn starch + green cardamom (CG), high-carbohydrate, high-fat (H), high-carbohydrate, high-fat + black cardamom (HB) or high-carbohydrate, high-fat + green cardamom (HG). CB, CG, HB and HG rats were fed with a basal C and H diet for the first 8 weeks of the protocol and for the next 8 weeks, these rats were treated with the same diet supplemented with 3% green or black cardamom (30 g/kg replacing 30 mL/kg water in the food). Powdered black cardamom was provided by Mr Parasuram Poudyal (Ranipool, Sikkim, India) and green cardamom was provided by Prof. Krishna Kumar (Coimbatore, Tamil Nadu, India). All H diet-fed rats were provided with 25% fructose added to the drinking water. C and H diets were prepared in our laboratory (Table 1) [19]. Measurements of body weight and food and water intakes were taken daily and feed efficiency (%) was calculated [20].

**Table 1:** Composition of corn starch (C) and high-carbohydrate, high-fat (H) diets.

Ingredient, g/kg	C	H
Corn starch	570.0	-
Powdered rat feed	155.0	155.0
HMW salt mixture	25.0	25.0
Fructose	-	175.0
Beef tallow	-	200.0
Condensed milk	-	395.0
Water	250.0	50.0
Energy, kJ/g	11.23	17.93

### 2.3. Oral Glucose Tolerance Test

At the end of the feeding protocol, rats were deprived of food overnight (12 h) and an oral glucose tolerance test was performed. Fructose-supplemented water in H, HG and HB groups was replaced with normal water during the food deprivation period. Basal blood glucose concentrations were measured in blood taken from the tail vein using Medisense Precision Q.I.D. glucose meter (Abbott Laboratories, Bedford, MA, USA). The rats were given 2 g/kg body weight of glucose as a 40% aqueous solution via oral gavage. Tail vein blood samples were taken at 30, 60, 90 and 120 min following glucose administration.

### 2.4. Systolic Blood Pressure

Systolic blood pressure was measured at 0, 8 and 16 weeks [19,20,21] under light sedation by intraperitoneal injection with Zoletil (tiletamine 15 mg/kg, zolazepam 15 mg/kg; Virbac, Peakhurst, New South Wales, Australia). Measurements were taken using MLT1010 Piezo-Electric Pulse Transducer (ADInstruments Australia, Bella Vista, New South Wales, Australia) and inflatable tail-cuff connected to a MLT844 Physiological Pressure Transducer (ADInstruments Australia, Bella Vista, New South Wales, Australia) and PowerLab data acquisition unit (ADInstruments Australia, Bella Vista, New South Wales, Australia).

### 2.5. Echocardiography

Echocardiographic examination using Hewlett Packard Sonos 5500, 12 MHz transducer was performed to assess the cardiovascular structure and function at 16 weeks two days after measurement of systolic blood pressure [19,20,21] under anaesthesia using intraperitoneal Zoletil (toletamine 15 mg/kg and zolazepam 15 mg/kg; Virbac, Peakhurst, New South Wales, Australia) and Ilium Xylazil (xylazine 15 mg/kg, intraperitoneal, Troy Laboratories, Smithfield, New South Wales, Australia) [19,20,21].

### 2.6. Isolated Heart Preparation

After 3 h of fasting, terminal anaesthesia was induced via intraperitoneal injection of pentobarbitone sodium (Lethabarb, 100 mg/kg) between 9 am and 5 pm. After heparin (Sigma-Aldrich Australia, Sydney, Australia) administration (200 IU) through the right femoral vein, blood (~6 mL) from the abdominal aorta was collected into heparinised tubes. Isolated Langendorff heart preparations were used to assess left ventricular function of the rats in all groups [19,20,21]. Isovolumetric ventricular function was measured by inserting a latex balloon catheter into the left ventricle of the isolated heart connected to a Capto SP844 MLT844 physiological pressure transducer and Chart software on a MacLab system (ADInstruments Australia and Pacific Islands, Bella Vista, New South Wales, Australia).

### 2.7. Aortic Contractility

Thoracic aortic rings (~4 mm in length) were suspended in an organ bath chamber filled with Tyrode physiological salt solution bubbled with 95% O_2_-5% CO_2_ and allowed to stabilise at a resting tension of 10 mN. Cumulative concentration-response curves (contraction) were obtained for noradrenaline (Sigma-Aldrich Australia) and cumulative concentration-response curves (relaxation) were obtained for acetylcholine (Sigma-Aldrich Australia, Sydney, New South Wales, Australia) and sodium nitroprusside (Sigma-Aldrich Australia, Sydney, New South Wales, Australia) following submaximal (70%) contraction to noradrenaline [19,20,21].

### 2.8. Body Composition Measurements

A Norland XR36 DXA (dual-energy X-ray absorptiometry) instrument (Norland Corp., Fort Atkinson, WI, USA) was used at the end of 16 weeks, 2 days after echocardiography and 2 days before terminal experiments, under anaesthesia with Zoletil (tiletamine 25 mg/kg and zolazepam 25 mg/kg) and Ilium Xylazil (xylazine 15 mg/kg) via intraperitonial injection. Scans were analysed using the manufacturer’s recommended software for use in laboratory animals (Small Subject Analysis Software, version 2.5.3/1.3.1; Norland Corp, Fort Atkinson, WI, USA) [19,20,21]. The precision error of lean mass for replicate measurements, with repositioning, was 3.2%. Visceral adiposity index (%) was calculated [19,20,21].

### 2.9. Organ Weights

The right and left ventricles were separated after perfusion experiments and weighed. Liver and abdominal fat were removed at the time of the heart removals for perfusion experiments and blotted dry for weighing. Perirenal, epididymal and omental fat were together weighed as abdominal fat. Organ weights were normalised relative to the tibial length at the time of their removal (in mg/mm). Immediately after weighing, the LV (left ventricular), liver and retroperitoneal fat were stored at −20 °C in 50-mL polypropylene centrifuge tubes for future analysis.

### 2.10. Histology

Two rats per group were taken exclusively for histological analysis. Soon after euthanasia, heart and liver were collected and fixed in 10% neutral buffered formalin for 3 days. The samples were then dehydrated and embedded in paraffin wax [19,20,21]. Thin sections (~7 µm) of left ventricle and liver were cut and stained with haematoxylin and eosin to study infiltration of inflammatory cells and for determining fat vacuoles in liver with a 20× objective using an Olympus BX51 microscope (Olympus, Melville, NY, USA). Further, heart left ventricular sections were stained with picrosirius red to study collagen deposition. Laser confocal microscopy (LSM 510 upright Confocal Microscope, Carl Zeiss, North Ryde, New South Wales, Australia) was used to determine the extent of collagen deposition in selected tissue sections.

### 2.11. Plasma Biochemistry

Blood was centrifuged at 5000× *g* for 15 min within 30 min of collection into heparinised tubes. Plasma was separated and transferred to Eppendorf tubes for storage at −20 °C before analysis. Plasma concentrations of total cholesterol, triglycerides, non-esterified fatty acids (NEFA), activities of plasma alanine transaminase (ALT), aspartate transaminase (AST) and alkaline phosphatase (ALP) were determined using kits and controls supplied by Olympus using an Olympus analyser (AU 400 Tokyo, Japan) [19,20,21]. Plasma insulin and leptin concentrations (ALPCO, Salem, NH, USA) were estimated using a commercial ELISA kit according to manufacturer-provided standards and protocols.

### 2.12. Statistical Analysis

All data are presented as mean ± standard error of mean (SEM). Results were tested for homogenous variance using Bartlett’s test and variables that were not normally distributed were transformed (using log 10 function) prior to statistical analyses. C, CG, CB, H, HB and HG groups were tested for effects of diet, treatment, and their interactions by 2-way Analysis of Variance (ANOVA). When the interaction and/or the main effects were significant, means were compared using Newman-Keuls multiple comparison post hoc test. Where transformations did not result in normality or constant variance, a Kruskal-Wallis nonparametric test was performed and *p* < 0.05 was considered significant. All statistical analyses were performed using GraphPad Prism version 6.00 for Windows (San Diego, CA, USA).

## 3. Results

### 3.1. Cardamom Analysis

Black cardamom contained 1,8-cineole as the major volatile constituent (>65%) while green cardamom contained α-terpinyl acetate (>72%) that was not present in black cardamom (Table 2). Black cardamom had an increased carbohydrate content but decreased fat content compared to green cardamom (Table 2).

**Table 2:** Cardamom analysis.

Variable	Green Cardamom	Black Cardamom
**Gas chromatography-mass spectrometry (GC-MS) area (%)**		
α-terpinyl acetate	72.73	-*
1,8-cineole	10.61	65.52
α-terpineol	0.86	3.29
limonene	0.38	3.59
α-pinene	1.50	2.84
β-pinene	0.23	3.43
**Composition**		
Energy (KJ/100 g)	1557	1477
Protein (% w/w)	10.8	9.3
Total fat (% w/w)	10.3	1.7
Moisture (% w/w)	12.2	9.4
Total carbohydrate (%)	58.4	73.9

Values are represented as mean of duplicate analysis; * not detected by GC-MS.

### 3.2. Metabolic Parameters

Food and water intake was decreased in H, HB and HG rats compared to C, CB and CG rats, respectively (Table 3). Cardamom supplementation did not alter food or water intakes in any group (Table 3). Green cardamom and black cardamom supplementation increased energy intake compared to C and H rats (Table 3). Feed conversion efficiency was increased in H rats compared to C rats but reduced by black cardamom (CB, HB) compared to green cardamom (CG, HG) (Table 3). Black cardamom groups showed decreased body weight gain and abdominal circumference compared to green cardamom and high-carbohydrate, high-fat diet-fed groups (Table 3). Body mass and visceral adiposity indices were reduced in HB rats only (Table 3). Bone mineral density was increased in H rats compared to C rats and normalised by black cardamom treatment only. Total body lean mass increased in H and HB rats compared to C, CB and CG rats. In HG rats, total body lean mass decreased but total body fat mass increased (Table 3). Black cardamom reduced total body fat in both CB and HB groups (Table 3). These changes in total body fat are consistent with abdominal fat measurements where black cardamom decreased and green cardamom increased abdominal fat pads (Table 3).

Plasma lipid concentrations were increased in H rats compared to C rats (Table 3). CB, CG and HB rats showed decreased plasma lipid concentration, in contrast to HG rats. Plasma insulin concentrations almost quadrupled in H rats compared to C rats (Table 3); these concentrations were decreased by both black and green cardamom. Oral glucose tolerance test showed improved glucose metabolism in C rats compared to H rats, while no significant changes were seen either with HG or HB treatment compared to H rats (Table 3).

### 3.3. Cardiovascular Structure and Function

Compared to C rats, H rats increased left ventricular weight and internal diameter in diastole as a sign of eccentric hypertrophy, without changes in relative wall thickness, increased stroke volume or cardiac output (Table 4). H rats also showed impaired cardiac function seen as increased systolic blood pressure and diastolic stiffness with increased diastolic, systolic and stroke volumes and decreased fractional shortening, developed pressure and rate of change of pressure (dP/dt) (Table 4). Green cardamom rats showed impaired cardiac function seen as decreased fractional shortening, increased wall stress, increased diastolic stiffness, decreased developed pressure and decreased dP/dt in HG rats. Additionally, diastolic, systolic and stroke volumes and cardiac output were elevated with green cardamom supplementation. HB rats showed normalised volumes, no signs of eccentric hypertrophy and normalised estimated left ventricular mass and hence improved cardiac function (Table 4). HB increased the heart rate and therefore the cardiac output. However, HB rats showed decreased systolic blood pressure, LV wet weight and diastolic stiffness constant when compared to H and HG rats; no significant changes were observed in CB rats (Table 4).

**Table 3:** Dietary intakes, body composition and anthropometrics, organ wet weights, changes in glucose tolerance test, plasma insulin and plasma biochemistry in corn starch (C), C + green cardamom (CG), C + black cardamom (CB), high-carbohydrate, high-fat (H), H + green cardamom (HG) and H + black cardamom (HB) diet-fed rats (*n* = 8 rats/group).

Variable	C	CG	CB	H	HG	HB	*p* values		
							Diet	Treatment	Interaction
Food intake (g/day)	33.8 ± 0.7 ^a^	35.1 ± 0.7 ^a^	34.6 ± 0.8 ^a^	26.9 ± 0.7 ^ab^	24.2 ± 0.5 ^b^	25.0 ± 0.6 ^ab^	<0.0001	0.55	0.0157
Water intake (mL/day)	23.9 ± 1.4 ^b^	36.8 ± 2.0 ^a^	27.2 ± 2.0 ^b^	26.6 ± 1.2 ^b^	28.0 ± 1.0 ^b^	35.0 ± 1.3 ^a^	0.65	<0.0001	<0.0001
Cardamom intake (g/day)	0.0 ± 0.0 ^c^	1.1 ± 0.0 ^a^	1.1 ± 0.0 ^a^	0.0 ± 0.0 ^c^	0.7 ± 0.0 ^b^	0.8 ± 0.0 ^b^	<0.0001	<0.0001	<0.0001
Cumulative energy intake from water (kJ)	0.0 ± 0.0 ^c^	0.0 ± 0.0 ^c^	0.0 ± 0.0 ^c^	6230 ± 700 ^b^	6,450 ± 350 ^b^	7,950 ± 490 ^a^	<0.0001	0.0554	0.0554
Cumulative energy intake from food (kJ)	21,640 ± 730	22,440 ± 690	22,150 ± 420	24,760 ± 670	24,680 ± 1290	24,890 ± 670	0.0001	0.88	0.85
Cumulative energy intake (kJ)	21,640 ± 730 ^b^	22,440 ± 690 ^b^	22,150 ± 420 ^b^	30,990 ± 730 ^a^	31,130 ± 1420 ^a^	32,840 ± 930 ^a^	<0.0001	0.40	0.51
Feed conversion efficiency (%)	1.6 ± 0.3 ^c^	3.1 ± 0.2^c^	1.3 ± 0.5 ^c^	8.4 ± 1.2 ^a^	8.1 ± 0.5 ^a^	4.8 ± 1.2 ^bc^	<0.0001	0.0029	0.0202
Initial body weight (g)	336 ± 3	338 ± 2	337 ± 2	336 ± 2	339 ± 2	336 ± 1	>0.99	0.59	0.89
Body weight at 8 weeks (g)	392 ± 7 ^b^	401 ± 10 ^b^	390 ± 5 ^b^	477 ± 15 ^a^	488 ± 14 ^a^	462 ± 12 ^a^	<0.0001	0.25	0.78
Body weight at 16 weeks (g)	417 ± 8 ^c^	435 ± 9 ^c^	409 ± 6 ^c^	561 ± 18 ^a^	574 ± 19 ^a^	506 ± 11 ^b^	<0.0001	0.0023	0.15
Body weight gained (8–16 weeks) (%) *	6.4 ± 1.3 ^b^	8.3 ± 1.2 ^b^	4.8 ± 1.2 ^b^	17.2 ± 1.3 ^a^	17.6 ± 1.1 ^a^	9.5 ± 2.4 ^b^	<0.0001	0.0003	0.0578
Visceral adiposity index (%)	4.5 ± 0.2 ^b^	4.2 ± 0.4 ^b^	4.4 ± 0.2 ^b^	7.2 ± 0.6 ^ab^	8.7 ± 0.5 ^a^	5.7 ± 0.4 ^b^	<0.0001	0.0057	0.0015
Abdominal circumference (cm)	20.0 ± 0.2 ^b^	21.5 ± 0.3 ^ab^	18.3 ± 0.2^c^	23.9 ± 0.4 ^a^	23.1 ± 0.3 ^a^	20.7 ± 0.3^b^	<0.0001	<0.0001	0.0011
Body mass index (kg/m^2^)	5.6 ± 0.2 ^b^	5.9 ± 0.2 ^b^	5.6 ± 0.1^b^	6.7 ± 0.2 ^a^	7.1 ± 0.1 ^a^	5.9 ± 0.1 ^b^	<0.0001	0.06	0.78
Bone mineral content (g)	12.7 ± 0.4 ^c^	13.1 ± 0.6 ^c^	12.0 ± 0.3 ^c^	16.0 ± 0.7 ^ab^	17.9 ± 0.4 ^a^	13.8 ± 0.6^c^	<0.0001	<0.0001	0.0223
Total body lean mass (g)	295 ± 7 ^b^	297 ± 7 ^b^	297 ± 7 ^b^	329 ± 9 ^a^	267 ± 10 ^c^	311 ± 6 ^b^	0.36	0.0019	0.0008
Total body fat mass (g)	100 ± 7 ^c^	112 ± 19 ^c^	77 ± 8 ^d^	203 ± 19 ^b^	270 ± 15 ^a^	140 ± 17 ^c^	<0.0001	<0.0001	0.0108
**Tissue wet weight (mg/mm)**									
Retroperitoneal adipose tissue	171 ± 10 ^c^	153 ± 16 ^c^	137 ± 9^c^	375 ± 48 ^b^	488 ± 43 ^a^	234 ± 28 ^c^	<0.0001	0.0002	0.0011
Epididymal adipose tissue	119 ± 8 ^b^	109 ± 12 ^b^	95 ± 9 ^b^	225 ± 23 ^a^	268 ± 28 ^a^	144 ± 15 ^b^	<0.0001	0.0008	0.0113
Omental adipose tissue	85 ± 7 ^b^	96 ± 10 ^b^	100 ± 8 ^b^	190 ± 19 ^a^	227 ± 27 ^a^	137 ± 13 ^b^	<0.0001	0.0282	0.0129
Liver	261.8 ± 10.1 ^b^	248.6 ± 11.3 ^b^	226.3 ± 4.7 ^c^	336.3 ± 12.3 ^a^	345.9 ± 10.4 ^a^	282.4 ± 11.0 ^b^	<0.0001	<0.0001	0.15
**Glucose metabolism and plasma biochemistry**									
OGTT-AUC (mmol/L·min)	659 ± 13 ^c^	722 ± 18 ^b^	715 ± 28 ^bc^	799 ± 9 ^a^	818 ± 19 ^a^	763 ± 7 ^a^	<0.001	0.0574	0.0364
Plasma insulin (μmol/L)	2.0 ± 0.2 ^b^	0.9 ± 0.1 ^b^	1.3 ± 0.2 ^b^	5.7 ± 1.3 ^a^	2.2 ± 0.5 ^b^	2.8 ± 0.5 ^b^	<0.001	0.0014	0.11
Plasma leptin (ng/mL)	3.3 ± 0.5 ^b^	5.3 ± 0.8 ^b^	1.9 ± 0.5 ^b^	7.9 ± 1.0 ^a^	9.1 ± 0.6 ^a^	3.4 ± 0.5 ^b^	<0.001	<0.0001	0.07
Plasma ALP (U/L)	131 ± 7 ^cd^	170 ± 13 ^cd^	113 ± 4	214 ± 18 ^b^	261 ± 23 ^a^	178 ± 15 ^c^	<0.001	0.0001	0.66
Plasma ALT (U/L)	30.1 ± 2.0 ^c^	34.8 ± 2.3 ^bc^	25.5 ± 0.8 ^c^	39.0 ± 3.8 ^a^	38.3 ± 1.6 ^bc^	35.4 ± 2.4 ^bc^	0.0003	0.0376	0.35
Plasma AST (U/L)	61.5 ± 1.5 ^b^	67.2 ± 3.5 ^b^	59.2 ± 1.7 ^b^	90.2 ± 5.6 ^a^	62.2 ± 2.0 ^b^	59.3 ± 1.2 ^b^	0.0024	<0.0001	<0.0001
Plasma total cholesterol (mmol/L)	1.8 ± 0.1 ^bc^	1.5 ± 0.1 ^c^	1.7 ± 0.1 ^c^	2.2 ± 0.1 ^a^	2.0 ± 0.1 ^ab^	1.8 ± 0.1 ^bc^	<0.001	0.0042	0.0487
Plasma triglycerides (mmol/L)	0.9 ± 0.1 ^b^	0.7 ± 0.1 ^b^	0.5 ± 0.1 ^b^	2.2 ± 0.4 ^a^	2.3 ± 0.3 ^a^	1.1 ± 0.2 ^b^	<0.001	0.0032	0.1
Plasma NEFA (mmol/L)	3.8 ± 0.6 ^bc^	2.1 ± 0.2 ^c^	2.2 ± 0.2 ^c^	6.6 ± 0.8 ^a^	6.2 ± 0.4 ^a^	4.1 ± 0.5 ^bc^	<0.001	0.0008	0.1

Each value is a mean ±Standard Error of the Mean (SEM). Means within a row with unlike superscripts differ, *p* < 0.05. ALP, alkaline phosphatase; ALT, aspartate transaminase; AST, aspartate transaminase; NEFA, non-esterified fatty acids. * Body-weight gain calculated as percentage of body weight increase from 8 weeks to 16 weeks (8 weeks).

**Table 4:** Changes in cardiovascular structure and function in C, CG, CB, H, HG and HB diet-fed rats (*n* = 8 rats/group).

Variable	C	CG	CB	H	HG	HB	*p* values		
							Diet	Treatment	Interaction
Heart rate (bpm)	268.8 ± 21.5 ^b^	236.0 ± 10.2 ^b^	299.4 ± 22.1 ^b^	352.6 ± 22.9 ^a^	255.1 ± 19.3 ^b^	317.5 ± 19.8 ^ab^	0.0164	0.0024	0.18
LVIDd (mm)	6.61 ± 0.27 ^b^	7.81 ± 0.16 ^a^	7.07 ± 0.18 ^b^	7.86 ± 0.36 ^a^	8.11 ± 0.16 ^a^	7.24 ± 0.26 ^b^	0.006	0.003	0.06
LVIDs (mm)	3.46 ± 0.12 ^b^	4.44 ± 0.18 ^a^	3.35 ± 0.16 ^b^	4.20 ± 0.16 ^a^	4.45 ± 0.28 ^a^	3.48 ± 0.24 ^b^	0.08	<0.0001	0.15
Fractional shortening (%)	53.0 ± 1.5 ^a^	43.3 ± 1.5 ^b^	55.3 ± 1.9 ^a^	51.4 ± 3.6 ^ab^	45.0 ± 3.3 ^b^	50.4 ± 2.3 ^a^	0.44	0.0016	0.42
(+)dP/dt (mmHg/S)	1298 ± 56 ^a^	1200 ± 63 ^a^	1265 ± 54 ^a^	842 ± 42 ^b^	883 ± 59 ^b^	1186 ± 61 ^a^	<0.0001	0.0045	0.0063
(−)dP/dt (mmHg/S)	−858. ± 38 ^a^	−687 ± 42 ^c^	−734 ± 27 ^cb^	−437 ± 38 ^d^	−532 ± 39 ^d^	−712 ± 38 ^cb^	<0.0001	0.0125	<0.0001
Diastolic stiffness (k)	22.7 ± 0.7 ^b^	23.6 ± 0.9 ^b^	22.2 ± 0.8 ^b^	28.5 ± 0.5 ^a^	27.3 ± 0.5 ^a^	24.1 ± 0.8 ^b^	<0.0001	0.0014	0.0286
Diastolic volume (μL)	313 ± 36 ^b^	504 ± 29 ^a^	377 ± 29 ^b^	530 ± 68 ^a^	562 ± 33 ^a^	408 ± 42 ^b^	0.0043	0.0037	0.07
Systolic volume (μL)	44 ± 5 ^b^	95 ± 11 ^a^	42 ± 6 ^b^	80 ± 9 ^a^	100 ± 16 ^a^	49 ± 10 ^b^	0.06	<0.0001	0.24
Stroke volume (μL)	269 ± 38 ^b^	409 ± 22 ^ab^	335 ± 28 ^b^	445 ± 63 ^a^	463 ± 31 ^a^	359 ± 37 ^b^	0.0092	0.06	0.11
Cardiac output (mL/min)	71.9 ± 13.0 ^b^	96.0 ± 5.5 ^b^	101.2 ± 10.6 ^b^	157.2 ± 23.7 ^a^	119.6 ± 16.1 ^ab^	115.4 ± 14.4 ^ab^	0.0017	0.88	0.0451
Estimated LV mass, Litwin (g)	0.80 ± 0.03 ^b^	1.04 ± 0.04 ^a^	0.90 ± 0.04 ^ab^	1.15 ± 0.07 ^a^	1.15 ± 0.06^a^	1.03 ± 0.07 ^a^	<0.0001	0.0364	0.06
LV + septum wet weight *	17.9 ± 1.7 ^b^	19.6 ± 0.7 ^ab^	16.6 ± 0.5 ^b^	21.9 ± 0.7 ^a^	22.8 ± 1.0 ^a^	18.0 ± 0.8 ^b^	0.0009	0.001	0.41
Relative wall thickness	0.56 ± 0.04	0.48 ± 0.01	0.53 ± 0.01	0.52 ± 0.03	0.48 ± 0.01	0.56 ± 0.02	0.86	0.0122	0.32
Systolic blood pressure (mmHg)	132 ± 3 ^c^	135 ± 2 ^c^	132 ± 2^c^	161 ± 3 ^a^	151 ± 2 ^b^	136 ± 1 ^c^	<0.0001	<0.0001	<0.0001
Systolic wall stress (mmHg)	80.4 ± 5.1 ^b^	111.5 ± 5.7 ^a^	72.9 ± 5.2 ^b^	105.5 ± 8.1 ^a^	112.8 ± 9.2 ^a^	81.2 ± 7.2 ^b^	0.047	<0.0001	0.22

Each value is a mean ± Standard Error of the Mean (SEM). LVIVd, left ventricular internal diameter in diastole; LVIDs, left ventricular internal diameter in systole; * in mg/mm tibial length. Means within a row with unlike superscripts differ, *p* < 0.05.

**Figure 1:**
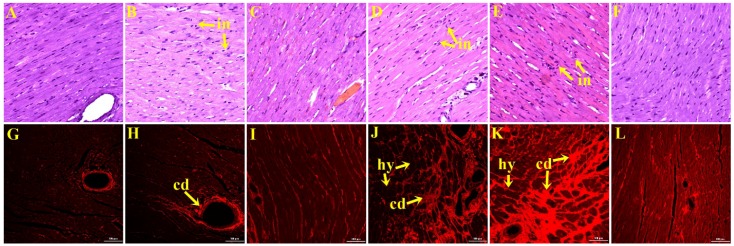
Haematoxylin and eosin staining of left ventricle (original magnification ×20) showing inflammatory cells (marked as “in”) as dark spots outside the cardiomyocytes in rats fed the C (**A**); CG (**B**); CB (**C**); H (**D**); HG (**E**) or HB (**F**) diet. Picrosirius red staining of left ventricular interstitial collagen deposition (original magnification ×20) in rats fed the C (**G**); CG (**H**); CB (**I**); H (**J**); HG (**K**) or HB (**L**) diet. Collagen deposition is marked as “cd” and hypertrophied cardiomyocytes are marked as “hy”. Corn starch (C), C + Green cardamom (CG), C + Black cardamom (CB), High-carbohydrate, high-fat (H), H + Green cardamom (HG) and H + Black cardamom (HB).

The LV of H rats showed greater infiltration by inflammatory cells (Figure 1D) as well as increased interstitial collagen deposition (Figure 1J) compared to C rats (Figure 1A,G, respectively). Black cardamom normalised the inflammatory state and markedly reduced collagen deposition in HB rats (Figure 1F,L, respectively). The reduction in LV fibrosis is consistent with the reduced diastolic stiffness constant in black cardamom rats. Green cardamom rats showed greater inflammatory cell infiltration (Figure 1B,E) and increased collagen deposition (Figure 1H,K) with hypertrophied cardiomyocytes in HG rats compared to H and HB rats and in CG rats compared to C and CB rats. No significant differences were observed between C and CB rats (Figure 1C,I).

**Figure 2:**
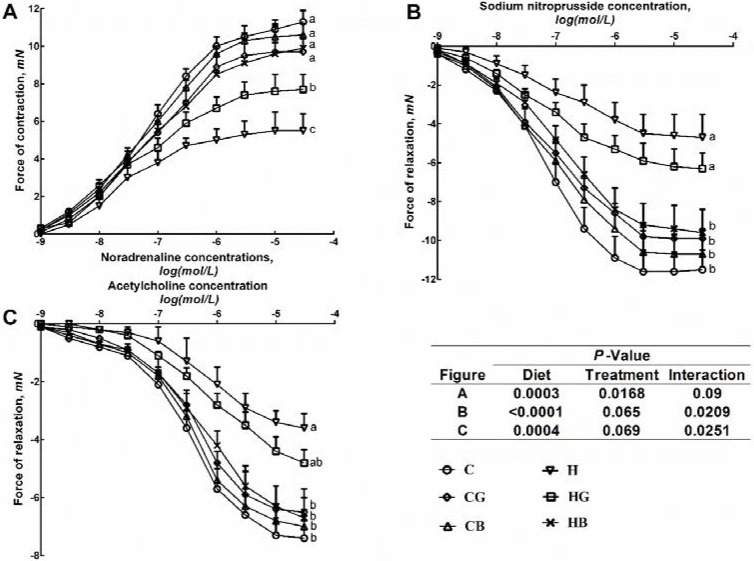
Cumulative concentration-response curves for noradrenaline (**A**); sodium nitroprusside (**B**) and acetylcholine (**C**) in thoracic aortic rings from rats fed the C, CB, CG, H, HB and HG diet. Data are shown as means ± standard error of the mean (SEM). Significantly different end-point means are indicated by different letters; *p* < 0.05 and *n* = 8/group. Corn starch (C), C + Green cardamom (CG), C + Black cardamom (CB), High-carbohydrate, high-fat (H), H + Green cardamom (HG) and H + Black cardamom (HB).

H rats showed diminished vascular contraction to noradrenaline in isolated thoracic aortic rings compared to C rats (Figure 2A). Additionally, H rats showed decreased smooth muscle-dependent and endothelium-dependent relaxant responses to sodium nitroprusside and acetylcholine, respectively (Figure 2B,C). Black cardamom rats showed increased vascular contraction to noradrenaline as well as increased smooth muscle-dependent and endothelium-dependent relaxant responses to sodium nitroprusside and acetylcholine, while HG rats failed to improve aortic function (Figure 2). These effects were associated with normalised systolic blood pressure in HB rats (Table 4).

### 3.4. Liver Structure and Function

In comparison to C rats, H rats had elevated plasma ALP, ALT and AST activities with increased liver weights. Black cardamom supplementation improved liver function, indicated by the decreased plasma activities of these enzymes. Green cardamom decreased plasma ALT and AST activity, but increased plasma ALP activity (Table 3).

**Figure 3:**
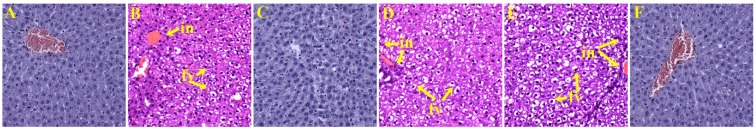
Haematoxylin and eosin staining of hepatocytes (original magnification ×20) showing inflammatory cells (marked as “in”) and hepatocytes with fat vacuoles (marked as “fv”) in rats fed the C (**A**); CG (**B**); CB (**C**); H (**D**); HG (**E**) and HB (**F**) diet.

H rats (Figure 3D) showed increased hepatic lipid deposition and inflammatory cell infiltration compared to C rats (Figure 3A). Black cardamom decreased macrovesicular steatosis and portal inflammation in HB rats (Figure 3F). In contrast, HG rats showed further increases in hepatic lipid deposition and inflammatory cell infiltration compared to H and HB rats (Figure 3E) and CG rats compared to C and CB rats (Figure 3B). No changes in tissue morphology, inflammatory cell infiltration or macrovesicular steatosis were seen in CB rats compared to C rats (Figure 3C).

## 4. Discussion

Rats fed on a diet with increased simple sugars such as fructose and sucrose together with increased saturated and *trans* fats developed abdominal obesity, hypertension, endothelial dysfunction and cardiac fibrosis together with an increase in ventricular stiffness, dyslipidaemia, liver inflammation, increased plasma lipid concentrations and impaired glucose tolerance [22,23,24,25]. These changes closely mimic human metabolic syndrome. Hence, we have used this rat model to investigate whether green or black cardamom can reverse these alterations in metabolic, cardiovascular and liver parameters.

There is no clear literature evidence that intervention with cardamom, either black or green, decreases the signs of the metabolic syndrome, although improvements in individual signs have been published. A green cardamom intake of 3 g/day lowered blood pressure in mildly hypertensive patients [26] and anti-inflammatory effects of green cardamom oil were measured in carrageenan-induced plantar oedema in male albino rats [13]. Black cardamom improved alcoholic fatty liver [27], lowered lipids in cholesterol diet-fed rabbits [28,29], improved glucose metabolism in fructose-fed rats [30] and decreased inflammation in carrageenan-induced paw oedema in rats [18]. It is assumed, but not proved, that the volatile oils are the major bioactive principles of cardamom. Further, cardamom contains unknown amounts of phenolic and flavonoid components that may have biological activity. The major constituent of volatile oil from black cardamom, 1,8-cineole, has potential effects in metabolic syndrome as this terpene dose-dependently reduced blood pressure in normotensive rats [31] and in nicotine-induced hypertensive rats [32], and also showed endothelium-dependent vasorelaxation in male Wistar rats [33]. Given the few studies reporting the therapeutic effects of green and black cardamom, the aim of this study was to determine the responses to chronic dietary supplementation of α-terpinyl acetate-containing green cardamom and 1,8-cineole-containing black cardamom in rats fed either low-fat, corn starch diet or a high-carbohydrate, high-fat diet as a model of metabolic syndrome. The responses to green and black cardamom were markedly different. Black cardamom reduced visceral adiposity; similarly, male Wistar rats fed with eucalyptus leaf extract containing high amounts of 1,8-cineole showed marked decreases in adipose fat mass [34]. This effect of black cardamom on adipose tissue could result from decreased infiltration of inflammatory cells in adipose tissue, as shown here in heart and liver. In contrast, green cardamom further increased visceral adiposity with decreased lean mass confirming that decreased muscle mass increases visceral adiposity [35].

Black cardamom improved liver function since the liver wet weight and activity of the liver plasma enzymes were lower than high-carbohydrate high-fat diet-fed rats and approximated those of corn starch diet-fed rats. Similarly, improved hepatic function was measured with black cardamom extract in alcohol-induced liver damage [27]. Black cardamom may protect the liver by increasing the expression of voltage-dependent anion channels that trigger the opening of mitochondrial membrane permeability transition pores [36]. Black cardamom also decreased plasma lipid concentrations and this action should decrease liver steatosis and insulin resistance, thus improving liver function.

In contrast, green cardamom has been reported to increase liver wet weight and increase plasma activities of ALT, AST and ALP, as markers of active liver damage [37]. In humans, green cardamom extract in Arabic coffee showed no effect on plasma liver enzyme activity [38]. From histological evidence, green cardamom further increased infiltration of inflammatory cells in the liver of high-carbohydrate, high-fat-fed rats; in contrast, green cardamom showed anti-inflammatory effects in acute carrageenan-induced plantar oedema in male albino rats [13]. Green cardamom did not change the increased plasma lipid profile in H rats, consistent with the presence of increased fat deposition in the liver.

Increased plasma free fatty acid concentrations and liver enzyme activities cause endothelial dysfunction leading to hypertension [39]. The high-carbohydrate, high-fat diet led to structural and functional changes in the heart. Cardiovascular abnormalities included increased left ventricular stiffness, increased relative wall thickness, reduced fractional shortening, reduced ejection fraction and increased estimated left ventricular mass [19]. Black cardamom normalised plasma free fatty acids concentrations, liver enzyme activities, thoracic aortic ring reactivity and cardiac structure and function. In patients with ischaemic heart disease, black cardamom (3 g/day) improved the plasma lipid profile and enhanced the fibrinolytic activity and antioxidant status, although cardiovascular parameters were not reported [40]. Green cardamom showed a smaller decrease in blood pressure, no changes in heart structure or thoracic aortic ring reactivity and increased inflammatory cell infiltration and collagen deposition, consistent with some changes in liver enzyme activities and insignificant changes in plasma free fatty acid concentrations. Green cardamom at 3 g/day for 12 weeks in mildly hypertensive subjects decreased blood pressure with no changes in plasma cholesterol and triglycerides [26], although liver function and heart structure were not measured. Green cardamom extract with Arabic coffee showed increased total cholesterol and LDL concentration with no effect on blood pressure [38].

The improved cardiovascular function in HB rats could be due to 1,8-cineole, as this compound decreased mean aortic pressure following increased values with hexamethonium, atenolol or methylatropine [31]. Further, 1,8-cineole (0.1 mg/kg/day) reduced hypertension induced by chronic nicotine administration and a higher dose (1 mg/kg/day) increased plasma nitrate concentrations [32]. Our results suggest that the improved vascular relaxant responses to acetylcholine following black cardamom led to decreased blood pressure. Black cardamom reduced left ventricular infiltration of inflammatory cells, local collagen deposition and left ventricular stiffness. Echocardiographic assessment of HB rats showed improved left ventricular function and decreased left ventricular dimensions. The total wet weight of the heart was also reduced. These results suggest that green and black cardamom produce different responses on cardiac structure and function, and on vascular responsiveness. Black cardamom improved the signs of metabolic syndrome, but the metabolic, cardiovascular and liver responses to the H diet were not improved by green cardamom in this study. Green cardamom (3 g/kg body weight) in mice showed altered energy metabolism, increased oxidative stress and morphological changes in heart structure [41]. Further, an increased dose of green cardamom in Arabic coffee may increase cardiovascular risk [38]. In this study, 30 g/kg food of either green or black cardamom was used to provide a daily dose of ~1.5 g/kg body weight, half the dose shown to increase oxidative stress in mice [41], which corresponds to ~20 g/d cardamom in a 70 kg human, based on body surface area comparisons between rats and humans [42]. This dose would seem too high to be obtained from the diet, suggesting that black cardamom may be useful in a combination with other functional foods to improve the signs of the metabolic syndrome in humans.

## 5. Conclusions

Black cardamom attenuated the signs of metabolic syndrome while green cardamom exacerbated adiposity, decreased liver function and worsened cardiovascular structure and function. However, green cardamom decreased plasma insulin and the liver enzymes, ALT and AST. These responses suggest that black cardamom containing 1,8-cineole may improve cardiac, hepatic and metabolic parameters, unlike green cardamom containing α-terpinyl acetate, which had no effect on heart and liver structure. Further investigations on these closely related terpenes will be necessary to understand their role in the improvement of the signs of metabolic syndrome, which is a limitation of the current study. Components other than the volatile oils such as phenolic and flavonoid constituents may also contribute to the differences in activity. In addition, black cardamom contains increased complex carbohydrates and this component may improve gastrointestinal function as dietary fibre decreases obesity [43,44].
